# A Case Report of Aggressive Angiomyxoma in the Buttock of a Female

**DOI:** 10.1002/ccr3.70822

**Published:** 2025-08-25

**Authors:** Aiwen Le, Zhibin Zheng, Tong Wu

**Affiliations:** ^1^ Department of Gynecology Shenzhen Nanshan People's Hospital, the Affiliated Nanshan Hospital of Shenzhen University Health Science Center Shenzhen Guangdong China; ^2^ Department of Radiology Shenzhen Nanshan People's Hospital, the Affiliated Nanshan Hospital of Shenzhen University Health Science Center Shenzhen Guangdong China; ^3^ Department of Pathology Shenzhen Nanshan People's Hospital, the Affiliated Nanshan Hospital of Shenzhen University Health Science Center Shenzhen Guangdong China

**Keywords:** aggressive angiomyxoma, buttock, imaging, layered sign, whorled sign

## Abstract

Aggressive angiomyxoma presents distinctive MRI features like “whirl sign” and “layering sign.” Laparoscopic combined with perianal incision can minimize bleeding and organ damage risks for pelvic‐extending tumors. Surgical excision with negative pressure drainage aids wound healing. Long‐term follow‐up is crucial to prevent recurrence.

## 1 Introduction

Steeper and Rosai first described nine cases of infiltrative, locally aggressive but nonmetastatic fibromyxoid soft tissue tumors in the pelvic and perineal regions of young females, naming them aggressive angiomyxomas (AAMs). The term “aggressive” refers to their potential infiltrative behavior [1]. The male‐to‐female ratio for AAM is 6.6:1 [2]. However, AAM lacks metastatic potential and exhibits nonmalignant histological features, resulting in a favorable prognosis [3]. Treatment primarily involves surgery, and despite wide excision with negative margins, there remains a possibility of local recurrence [4]. AAM predominantly occurs in the genital, perineal, and pelvic regions, with a few cases involving the buttocks, posterior thigh, and inguinal regions [5]. This case occurred in the right buttock. An analysis of the clinical characteristics of this case can provide valuable insights for diagnosis and treatment. The patient signed an informed consent form.

## 2 Case History/Examination

**FIGURE 1:**
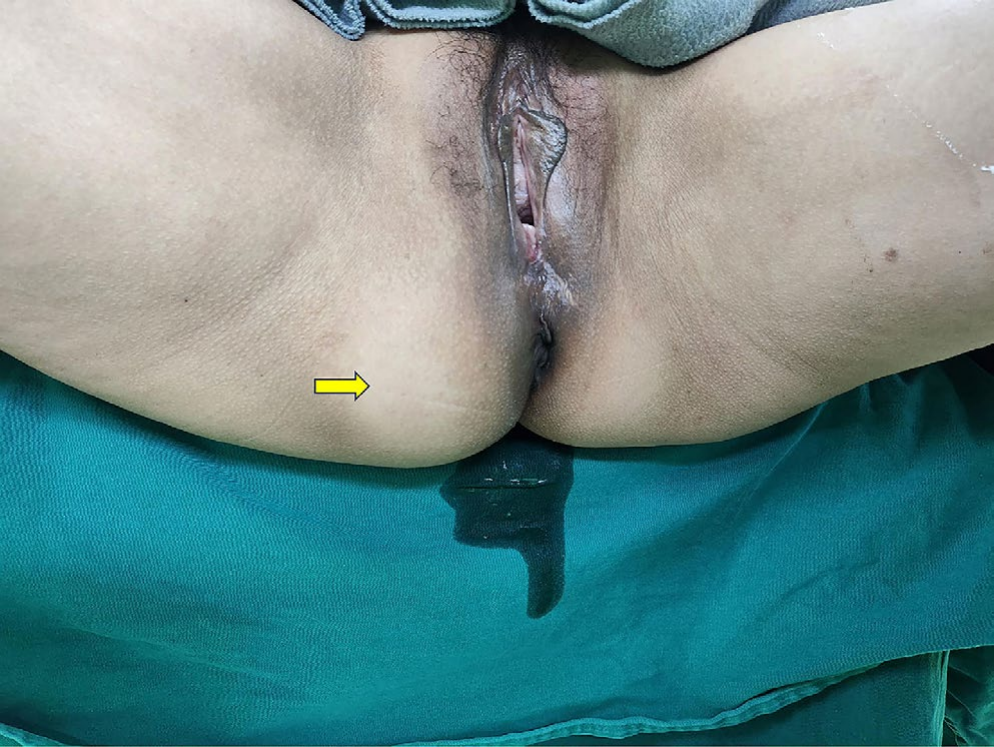
The right buttock appearance. Local protrusion of about 2 cm on the right buttock.

**FIGURE 2:**
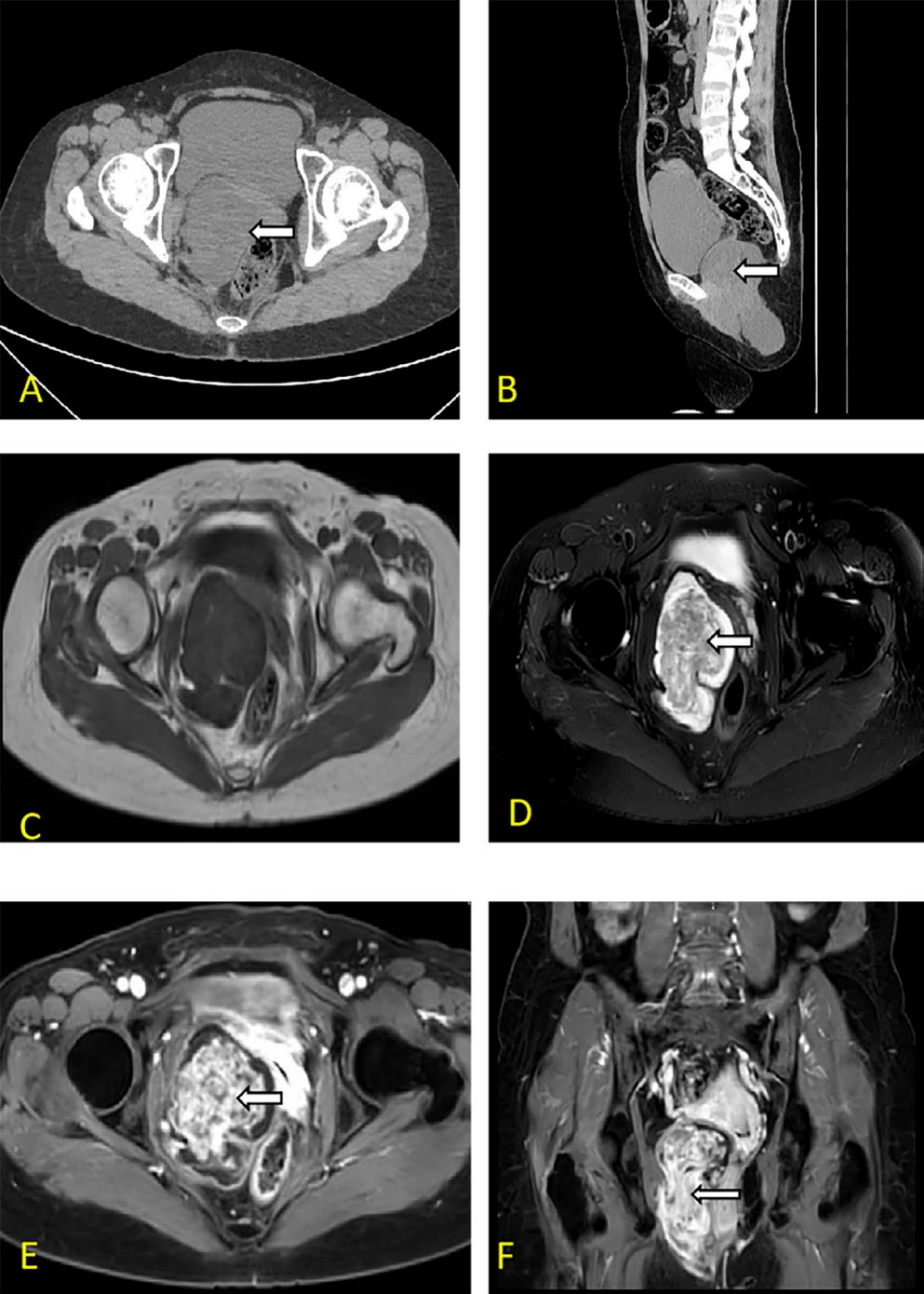
Patient's CT and MRI image. A large irregular mass on the right side of the pelvic floor extended through pelvic diaphragm into the ischiorectal fossa, displacing the rectum, uterus and vagina without invading. (A) Unenhanced CT image showed large pelvic mass being hypo‐attenuated in relation to surrounding muscular tissue. (B) Sagittal reconstruction image of CT scan showed a pedunculated soft tissue mass extending from the pelvic down to the subcutaneous soft tissue of the right buttock. (C) Axial T1‐weighted image showed a large pelvic floor mass was isointense and had a clear border to other tissue. (D) Axial fat‐saturated T2‐weighted imaging demonstrated a high‐signal intensity mass with inhomogeneity can be seen, and within it, there are streaky low‐signal intensity shadows running in bundles, showing its “layered” or “whirlpool” arrangement characteristics (white arrow). (E) Axial T1‐weighted post‐contrast image showed on the T2WI fat‐suppressed sequence, a high‐signal intensity mass with inhomogeneity can be seen, and within it, there are streaky low‐signal intensity shadows running in bundles, showing its “layered” or “whirlpool” arrangement characteristics. (F) Coronal T1‐weighted post‐contrast image revealed its “layered” or “whirlpool” arrangement characteristics (white arrow).

The patient, a 50‐year‐old married woman with children, was admitted to the hospital because of the “discovery of swelling in the right buttock for over 2 months.” Gynecological examination revealed a hard nodule in the right pelvic area, approximately 5 cm in diameter, with good mobility. There was a local protrusion on the right buttock of approximately 2 cm (Figure 1). Auxiliary examination: Evaluation of an MRI scan performed at another hospital on December 6, 2024, revealed a cystic–solid mass anterior to the sacrum, with the upper edge reaching the pelvic cavity and the lower edge located beside the anal canal, measuring 65 × 143 mm. A clump of equal T1 and short T2 abnormal signals was observed at the fundus of the uterus, with a maximum cross‐section of approximately 58 × 42 mm, suggesting the following: (1) a presacral space‐occupying lesion, such as a teratoma; (2) uterine fibroids; or (3) a cystic lesion in the left adnexal area. Levels of tumor markers such as human epididymis protein 4 (HE4), neuron‐specific enolase, CA125, CA199, and CA153 were within normal limits. Findings from a contrast‐enhanced pelvic MRI scan performed at our hospital indicated the following: (1) a cystic‐solid mass on the right side of the uterus and rectum that was considered to be of vascular origin, not excluding a fibrotic origin; and (2) multiple uterine fibroids (Figure 2).

## 3 Differential Diagnosis, Investigations, and Treatment

**FIGURE 3:**
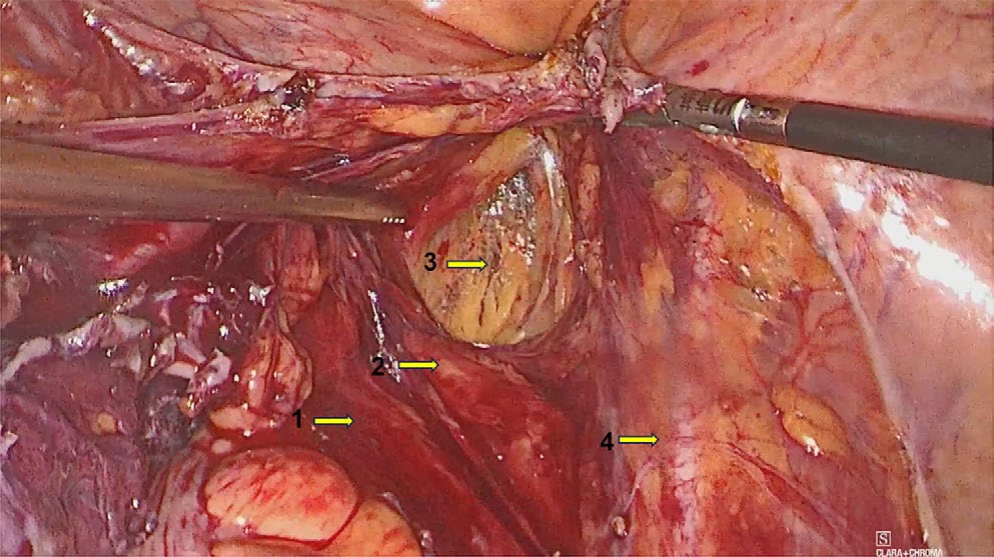
Right obturator fossa under laparoscopy. No mass was found in the right retroperitoneum under laparoscopy. 1: Right ureter, 2: Right internal iliac artery, 3: Right obturator, 4: Right external iliac artery.

**FIGURE 4:**
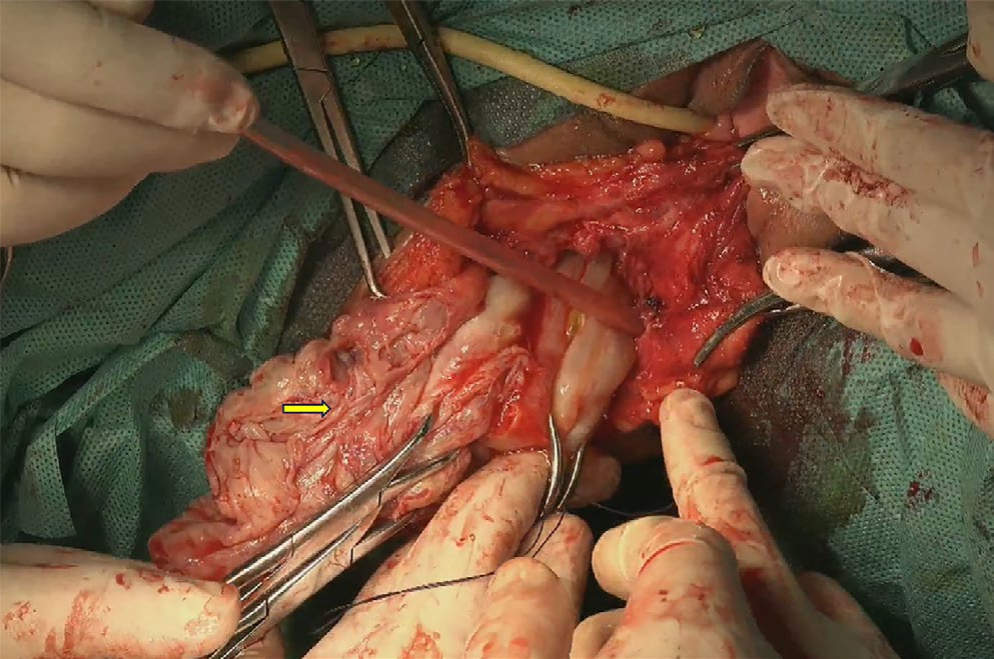
Resection of the mass near the anus.

**FIGURE 5:**
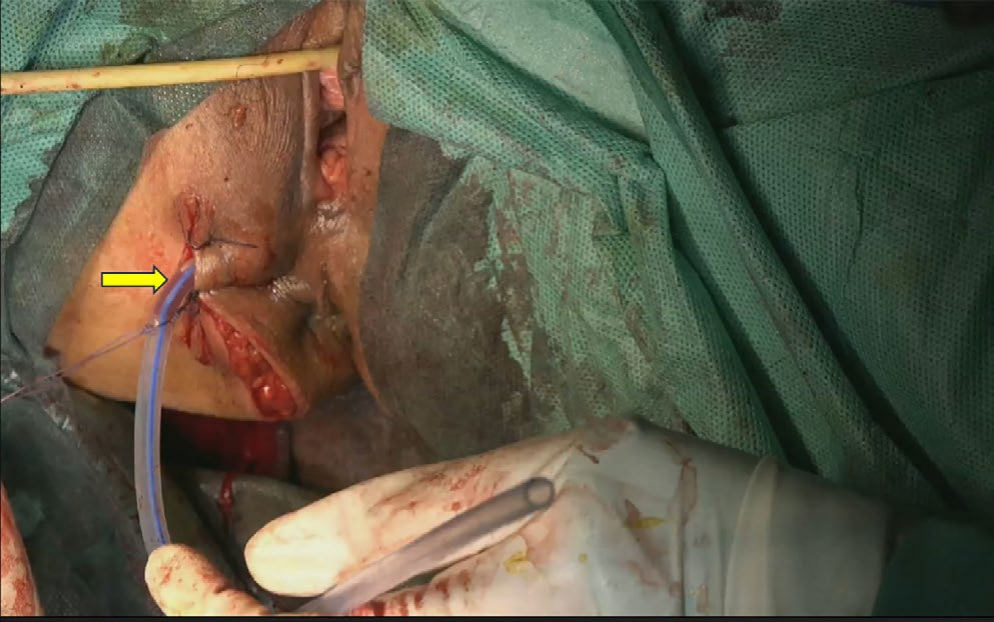
No. 18 porous negative pressure drainage tube for hip incision.

Preoperative Diagnosis: Presacral mass, nature undetermined, suspected vascular tumor. The preoperative plan was to perform laparoscopy combined with a perianal incision. On December 13, 2024, a laparoscopic total hysterectomy + bilateral adnexectomy + pelvic lesion resection (buttock mass resection via perianal incision) was performed. Laparoscopic exploration revealed a hard nodule with a diameter of 5 cm on the posterior wall of the uterus. The right posterior peritoneum was opened, and no mass was found (Figure 3). The mass was resected through the perianal incision and measured 65 × 110 mm (Figure 4). An 18‐gauge multihole negative pressure drainage tube was placed in the buttock incision (Figure 5).

## 4 Conclusion and Results (Outcome and Follow‐Up)

**FIGURE 6:**
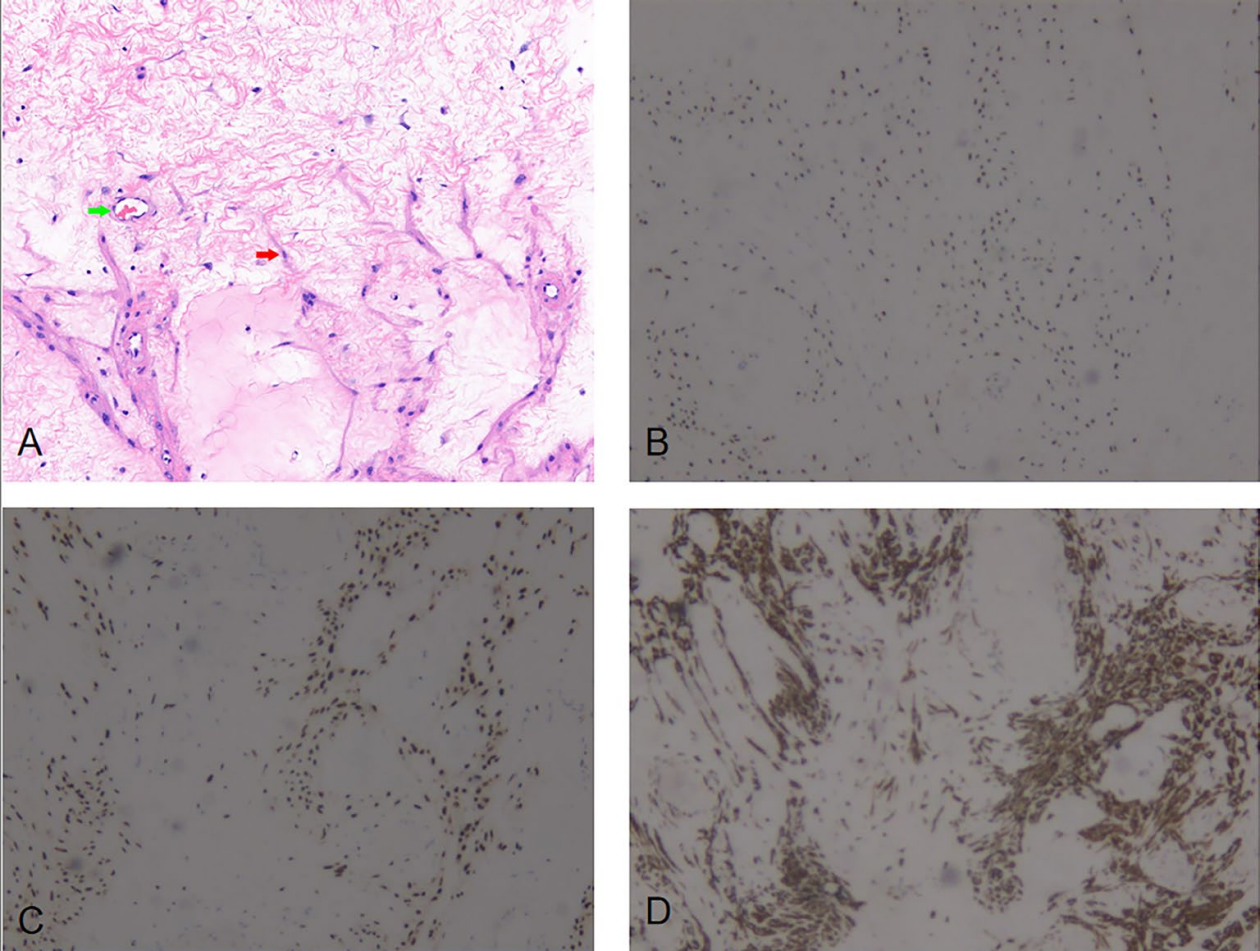
Patient's pathology. (A) was H&E, (B–D) were IHC and magnification was 5 × 10, brown was positive. (A) The cellular density was low, primarily composed of morphologically consistent stellate, oval, or short spindle‐shaped tumor cells (green arrow), distributed within a myxoid stroma. The tumor contains dilated thin‐walled blood vessels (red arrow). (B) ER; (C) PR; and (D) Desmin.

Postoperative pathology revealed that the tumor was composed of a small number of oval, spindle, and star‐shaped cells scattered in a large amount of sparse mucinous stroma. The stroma usually contains medium‐sized blood vessels with walls of varying thickness, some of which may show hyaline degeneration with Desmin(+), ER(+), PR(+). (Figure 6). The tumor lacked a capsule and exhibited some soft tissue infiltration, interspersing among muscles, nerves, and adipose tissue. The incision healed well, and the patient was discharged. Post‐discharge Follow‐up Content: The patient's condition is good. The incision is painless. One month after surgery, she recovered all daily activities and routines. At the 5‐month follow‐up ultrasound, no pelvic mass was detected.

In summary, when patients present with buttock masses, comprehensive imaging of the pelvic and abdominal regions should be performed. If imaging reveals a buttock mass with MRI features such as the “whorled sign” or “layered sign,” suspicion of AAM should be raised, and a multidisciplinary consultation should be conducted preoperatively. For masses protruding into the pelvic cavity, a laparoscopic approach combined with a perianal incision can reduce the risk of bleeding and organ damage. The use of a multihole negative pressure drainage tube in the buttock can promote wound healing. Long‐term follow‐up is essential postoperatively.

## 5 Discussion

AAM often presents as a slow‐growing polypoid skin lesion and must be differentiated from angiomyoblastoma, myxoid neurofibroma, myxoma, spindle cell lipoma, myxoid liposarcoma, leiomyosarcoma, and myoid rhabdomyosarcoma [6]. AAM is characterized by myofibroblastic differentiation of spindle or stellate cells, divided by a myxoid stroma and vascular components. AAM features vascular structures of varying sizes, abundant pink cytoplasm, and mild nuclei [7]. In general, there is no cellular atypia or coagulative tumor cell necrosis. AAMs are positive for desmin, smooth muscle actin, muscle‐specific actin, vimentin, CD34, estrogen, and progesterone receptors expression but negative for expression [8]. It was immunohistochemical staining Desmin(+), ER(+), PR(+) in this case. Diagnosis can be challenging when AAM is accompanied by hyalinized vascular deposits or a neurofibroma‐like appearance [9]. The diameter ranges from 1 to 60 cm, but the chance of recurrence does not depend on size [10]. In this case, the patient's buttock mass was soft enough to touch and was located in the medial buttock region, with a diameter of 11 cm. The preoperative diagnosis was unclear, with imaging findings suggesting a tumor of vascular origin and not excluding malignancy. Aggressive angiomyxomas typically present as large, well‐defined, multicompartmental soft‐tissue tumors. These tumors are iso‐ or hypointense to muscle on T1‐weighted images and heterogeneously hyperintense on T2‐weighted images. Furthermore, these tumors are hyperintense on DW images (b = 1000 s/mm) but hypointense on apparent diffusion coefficient mapping images. These masses exhibit marked heterogeneous enhancement following Gd‐DTPA administration. AAMs are predominantly composed of a mucinous matrix interspersed with mucoprotein components, and the mixture of these two elements results in a swirl or laminated appearance of signal intensity on T2‐weighted images; enhancement has also been reported as a characteristic feature. Postoperative pathology reveals oval, spindle, and stellate cells, with partial vascular hyalinization.

Radical surgery with negative‐margin resection remains the gold standard for treatment. Angiomyxoma exhibits low mitotic activity, and radiotherapy and chemotherapy are less commonly used [11]. Most patients' tumors are positive for estrogen and progesterone receptors and respond to hormonal therapies such as gonadotropin‐releasing hormone (GnRH) agonists, raloxifene, and tamoxifen. Aromatase inhibitors have been used preoperatively to reduce tumor size [12]. However, these GnRH agonists can cause menopausal‐like symptoms, such as osteoporosis, and tumor regrowth after discontinuation of these drugs limits their use [13]. Because the patient has already undergone bilateral oophorectomy, hormone therapy is no longer necessary. In this case, since no critical organs around the tumor were involved, surgical resection was considered feasible; given the significantly reduced hormone production following bilateral ovarian removal, these medications were not administered. Given the location of the mass in the buttock, a laparoscopic approach combined with a perianal incision was adopted. The use of a negative pressure drainage tube reduces the risk of buttock incision infection. The recurrence rate at the same site as the initial resection ranges from 36% to 72% [11, 14]. Most cases recur within 2 years; however, recurrence can occur months or even up to 20 years after initial treatment [15]. Therefore, all patients should undergo long‐term follow‐up, potentially extending beyond 20 years.

## Author Contributions

**Aiwen Le:** conceptualization, funding acquisition, investigation, project administration, supervision, writing – original draft, writing – review and editing. **Zhibin Zheng:** data curation. **Tong Wu:** data curation, investigation.

## Consent

The authors have obtained written informed consent from the patient.

## Conflicts of Interest

The authors declare no conflicts of interest.

## Data Availability

The data that support the findings of this study are available from the corresponding author upon reasonable request.
